# Analysis of Endogenous Peptides Released from Osteoarthritic Cartilage Unravels Novel Pathogenic Markers*[Fn FN2]

**DOI:** 10.1074/mcp.RA119.001554

**Published:** 2019-07-27

**Authors:** Patricia Fernández-Puente, Lucía González-Rodríguez, Valentina Calamia, Florencia Picchi, Lucía Lourido, María Camacho-Encina, Natividad Oreiro, Beatriz Rocha, Rocío Paz-González, Anabel Marina, Carlos García, Francisco J. Blanco, Cristina Ruiz-Romero

**Affiliations:** ‡ Proteomics Unit-PBR2-ProteoRed/ISCIII, Grupo de Investigación de Reumatología (GIR). Instituto de Investigación Biomédica de A Coruña (INIBIC), Complexo Hospitalario Universitario de A Coruña (CHUAC), SERGAS. As Xubias, 84, 15006 A Coruña, Spain.; § Agrupación Estratégica CICA – INIBIC, Universidade da Coruña, 15071 A Coruña, Spain.; ¶ Centro de Biología Molecular Severo Ochoa, CSIC. Nicolás Cabrera, 1, 28049 Madrid, Spain.; ‖ Departamento de Medicina, Fisioterapia y Ciencias Biomédicas. Universidade da Coruña, 15006 A Coruña, Spain.; ** RIER-RED de Inflamación y Enfermedades Reumáticas, INIBIC-CHUAC, As Xubias 84, 15006 A Coruña, Spain; ‡‡ CIBER-BBN Instituto de Salud Carlos III INIBIC-CHUAC As Xubias 84, 15006 A Coruna, Spain

**Keywords:** peptidomics, biomarker: diagnostic, secretome, targeted mass spectrometry, extracellular matrix, cartilage, neopeptides, osteoarthritis

## Abstract

Osteoarthritis (OA) is a pathology characterized by the loss of articular cartilage. In this study, we performed a peptidomic strategy to identify endogenous peptides (neopeptides) that are released from human osteoarthritic tissue, which may serve as disease markers. With this aim, secretomes of osteoarthritic and healthy articular cartilages obtained from knee and hip were analyzed by shotgun peptidomics. This discovery step led to the identification of 1175 different peptides, corresponding to 101 proteins, as products of the physiological or pathological turnover of cartilage extracellular matrix. Then, a targeted multiple reaction monitoring-mass spectrometry method was developed to quantify the panel of best marker candidates on a larger set of samples (*n* = 62). Statistical analyses were performed to evaluate the significance of the observed differences and the ability of the neopeptides to classify the tissue. Eight of them were differentially abundant in the media from wounded zones of OA cartilage compared with the healthy tissue (*p* < 0.05). Three neopeptides belonging to Clusterin and one from Cartilage Oligomeric Matrix Protein showed a disease-dependent decrease specifically in hip OA, whereas two from Prolargin (PRELP) and one from Cartilage Intermediate Layer Protein 1 were significantly increased in samples from knee OA. The release of one peptide from PRELP showed the best metrics for tissue classification (AUC = 0.834). The present study reveals specific neopeptides that are differentially released from knee or hip human osteoarthritic cartilage compared with healthy tissue. This evidences the intervention of characteristic pathogenic pathways in OA and provides a novel panel of peptidic candidates for biomarker development.

## Abstract (precis)

Osteoarthritis (OA) is a disease primarily characterized by the loss of cartilage extracellular matrix. We have followed a peptidomic strategy to identify endogenous peptides (neopeptides) released from healthy and OA human knee and hip articular cartilage, which may serve as disease markers. This study provides a comprehensive neopeptidomic profile of healthy and diseased tissues, and the identification and validation of a panel of eight endogenous peptides that are differentially released from the extracellular matrix because of the pathogenic process.

## Graphical Abstract

**Figure:**
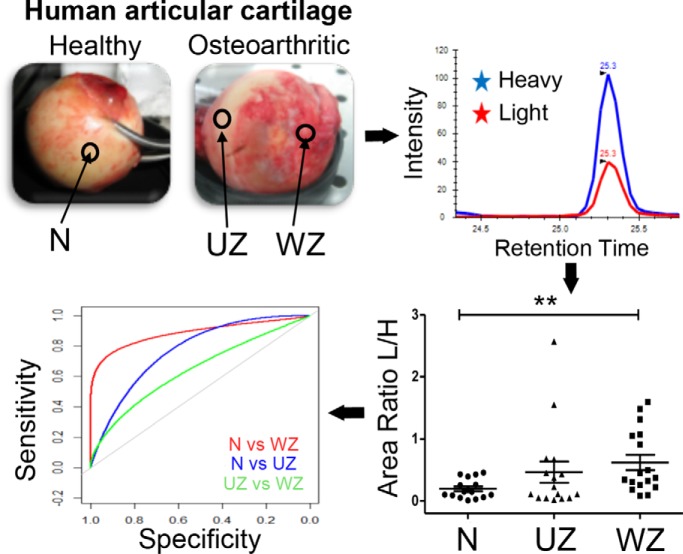

**Highlights**

- Shotgun identification of neopeptides released from osteoarthritic cartilage.
- Specific endogenous peptides from the cartilage ECM are measured by MRM.
- Identification of neopeptides differentially generated from diseased tissue.
- The peptide DSNKIETIPN shows the best metrics as biomarker of OA cartilage.

Osteoarthritis (OA)^1^ is the most common arthritic disease (1). It is already one of the ten most disabling pathologies in developed countries, becoming even more prevalent as the population ages and obesity rates rise. This disease is clinically silent in most patients in their early stages; thus, the deterioration of cartilage (one of the hallmarks of OA) is already extensive at the time of diagnosis. Therefore, the development of strategies for early diagnosis and accurate monitoring of disease progression is among the major research goals in OA.

OA is characterized by the loss of structural constituents from the extracellular matrix (ECM) of articular cartilage (2). The ECM maintains and supports chondrocytes within their natural physicochemical micro-environment (3), and the degradation and release of cartilage proteins can vary according to the stage of the disease process. Degradation of cartilage ECM proteins by specific proteinases is one of the main factors involved in OA pathology that contributes to disease progression. Several proteases have been extensively described as responsible for the degradation of cartilage ECM proteins in OA such as Metalloproteinases (MMPs), A Disintegrin And Metalloproteinase with Thrombospondin motifs (ADAMTSs), cathepsins, calpains and caspases, among others (4). Therefore, the presence of cartilage-characteristic proteins and their degradation products in proximal or peripheral body fluids, such as synovial fluid, blood or urine has been extensively evaluated to assess their biomarker usefulness. As examples confirming this hypothesis, the increase of the type II collagen fragment CTXII in urine has demonstrated a predictive value for disease progression (5, 6), and elevated levels of Cartilage Oligomeric Matrix Protein (COMP) in serum are correlated with the presence of OA and disease severity (7). Altogether, the ability to detect biomarkers of cartilage degradation and/or inflammation in biological samples, such as cartilage, serum, urine or synovial fluid, may be helpful to improve OA diagnosis, predict its progression and/or develop effective therapeutic strategies. In this area, proteomics has demonstrated to be a powerful tool for biomarker discovery in OA research (8, 9).

The term “peptidomics” was introduced as a branch derived from proteomics to define the quantitative and qualitative analysis of endogenous peptides (also named neopeptides) in biological samples, primarily by liquid chromatography (LC) or biochip platforms coupled to various forms of mass spectrometry (MS) (10). A specific neopeptide can be released from a protein because of the existence or progression of a specific disease. Therefore, peptidomics has been appealing for biomarker studies because the knowledge that is generated may present a dynamic view of health status: peptides are created by a complex and fluid interaction of proteases, activators, inhibitors and protein substrates (11). Because of many difficulties, biomarker discovery of endogenous peptides in complex samples is challenging and require systematic peptide extraction to achieve successful analysis (12).

In OA, previous studies have been focused on the influence that soluble mediators (such as specific cytokines and proteases, as mentioned above) or other external stimuli could have on cartilage degradation. For instance, diverse peptidomic studies have been conducted under the action of well-known OA-related proteinases (13), or inducers of cartilage degradation such as mechanical damage or proinflammatory cytokines (14, 15). In this work, we aimed to characterize the profile of neopeptides present in conditioned media (secretomes) from human articular cartilage, and quantitatively compare these profiles between healthy and osteoarthritic tissues. This would allow not only to identify potential neopeptide biomarker candidates, but also to foster the understanding of specific protease pathways that may be relevant for cartilage ECM destruction, which is the hallmark pathogenic process in OA.

## EXPERIMENTAL PROCEDURES

### 

#### 

##### Experimental Design and Statistical Rationale

The experimental design and statistical rationale for each of the experiments conducted in this work will be described more in detail in each subsection. The discovery phase (shotgun proteomics) was performed on six secretome samples (two biological replicates per condition), without technical replication. The development of targeted proteomics methods was performed using 34 samples and the validation was performed on 62. The size of the groups allowed to average out biological variations that were calculated during method development.

##### Human Articular Cartilage Specimens

Articular cartilage for the proteomic analysis was obtained either from hip femoral heads or knee condyles of patients with OA undergoing hip or knee replacement, and donors with no history of joint disease (N). None of them were *post mortem*. All tissue samples were provided by the Tissue Bank and the Autopsy Service at Hospital Universitario de A Coruña. The study was approved by the local Ethics Committee (Galicia, Spain). OA patients were diagnosed following the criteria determined by the American College of Rheumatology (16). Cartilage samples from 4 patients were used for the shotgun analysis (2 OA and 2 N), from additional 21 were employed for MRM development (13 OA and 8 N), and from further 40 in the validation studies (22 OA and 18 N).

The demographic characteristics of the donors are detailed in Table I.

##### Histological-Histochemical Grading of Cartilage

A modified Mankin scoring (17) was employed for the histopathological classification of the severity of lesions on all the cartilage samples employed in this work. Briefly, tissue sections (4 μm) were stained with hematoxylin and eosin to evaluate cellular architecture, and with toluidine blue and safranin O/fast green to visualize the matrix proteoglycan content. Three different aspects of the score were determined and summed up: cartilage structure (0–7 points), cellular abnormalities (0–2 points) and matrix staining (0–4 points), leading to a scale that ranges between 0 and 13. The Mankin score 0–2 represents normal cartilage, 3–5 superficial fibrillation, 6–7 moderate cartilage destruction, 8–10 severe damage of cartilage, and over 10 complete loss of cartilage.

##### Cartilage Explants Culture and Obtention of Secretomes

Tissue explants were obtained from the dissection of N and OA hip and knee cartilages as described previously (18). Among the OA samples, we differentiated the wounded zones (WZ) from those corresponding to the area adjacent to the lesion, or unwounded zones (UZ). The development of targeted proteomics methods was performed using 8 N, 13 WZ and 13 UZ, whereas the validation was performed on 62 cartilage samples, 18 N, 22 WZ and 22 UZ. The size of the groups allowed to average out biological variations that were calculated during method development.

Three 6-mm explants were cut from each zone/condition using a sterile biopsy punch. After extensive washes with PBS (Oxoid, Thermo Fisher Scientific, MA, USA), the discs were placed into 96-well plates (one disc/well), containing 200 μl of serum-free DMEM (Gibco, Thermo Fisher Scientific) supplemented with 100 units/ml penicillin and 100 μg/ml streptomycin to avoid contamination. Plates were incubated overnight at 37 °C, 5% CO_2_. The collection timeline of conditioned media (secretomes) was optimized based on our previous experience (18) and after appraising representative peptidomic profiles along 6 days. Secretomes from day 1 were discarded and replaced with fresh medium. Then, they were collected at days 2 and 5 from each explant culture. Protein concentrations were determined by the Bradford assay, and the samples were frozen at −80 °C until processing.

##### Secretome Processing

Secretomes from the same donor and condition (WZ, UZ or N) collected at days 2 and 5 were mixed together in a total volume of 1200 μl. The endogenous peptides were concentrated by ultrafiltration using Amicon Ultra-4 devices (10 kDa MWCO, Merck Millipore). The resulting eluted volumes (fractions comprising peptides of < 10 kDa), were dried in a vacuum concentrator (Savant SpeedVac, Thermo Fisher). The samples were cleaned twice prior to LC-MS/MS analysis, first by homemade Stage Tips containing six C18 Solid Phase Extraction Disks (Empore, Sigma) using a centrifuge (Hermle, Germany) at low revolutions per minute (rpm), and then by commercial C18 NuTips (Glygen), both using LC/MS-grade chemicals (Fisher Scientific, MA). For the conditioning steps using Stage Tips, methanol (2*100 μl) was used, followed by 80% acetonitrile (ACN) in 0.5% trifluoroacetic acid (TFA) (2*100 μl) and finally 0.5% TFA (3*100 μl). The secretome cartilage sample was reconstituted in 75 μl 0.5% TFA and loaded onto the six C18 disks twice for achieving maximum peptide binding. The bound sample was then washed four times (two with 100 μl 0.5% TFA and two with 100 μl 0.5% formic acid (FA)). Then, the sample was eluted twice with 80% ACN in 0.5% FA and dried using a speed vacuum concentrator (Themo Fisher). The second clean-up using NuTips, was performed according to the manufacturer's recommendation. Briefly, the resin was conditioned using 50 μl of 80% ACN, 0.1% TFA five times and 5 additional times with 50 μl 0.1% TFA. The dried sample from the first clean-up was reconstituted in 30 μl 0.1% TFA and passed through the resin 50 times. The unbound sample was washed six times with 40 μl 0.1% TFA and seven times with 40 μl 0.1% FA. Finally, the sample was eluted twice with 20 μl 80% ACN in 0.1% FA, aspirating and disposing the buffer for 50 times in each elution. The resulting 40 μl were dried in a vacuum concentrator and stored at −20 °C until reconstitution for LC-MS/MS analysis.

##### Preparation of Samples for MRM Quantification

Heavy stable synthetic isotope-labeled peptides (SIS peptides, crude purity) were purchased from Thermo Scientific. These peptides incorporated a fully atom labeled ^13^C and ^15^N isotopes at the different amino acids (labeled position; mass shift) as Alanine (^13^C_3_,^15^N-Ala; +4 Da) (A), Proline (^13^C_5_,^15^N-Pro; +6 Da) (P), Valine (^13^C_5_,^15^N-Val; +6 Da) (V), Leucine (^13^C_6_,^15^N-Leu; +7 Da) (L), Lysine (^13^C_6_,^15^N_2_-Lys; +8 Da) (K), or Arginine (^13^C_6_,^15^N_4_-Arg; +10 Da) (R). Individual stocks of each peptide ranging from 2.25–19.5 μg/μl were made. Then, equal volumes of each peptide were mixed to make the standard mixture solution. Finally, a dilution of 1/5000 of this mixture was made as the stock solution in a concentration range of 1.78–17.6 pmol/μl of each peptide. Aliquots were kept at −20 °C. The processed cartilage secretome samples used to develop the targeted MRM method were reconstituted in 7 μl of buffer A (0.1% FA in 5% ACN), whereas the set of samples used for the validation was reconstituted in 7 μl of the peptide stock solution. A single injection was used for each sample because of the low concentration of peptides in the matrix. A blank solvent sample was injected between samples to avoid carry over in the nanoLC-MRM system. The linearity, LOD and LOQ were not performed because of the low concentration of the cartilage samples matrix.

##### Discovery Phase Analysis by Shotgun LC/MS-MS

Six secretome desalted samples (*n* = 6, 2 N, 2 UZ, 2 WZ) were dried, resuspended in 10 μl of 0.1% FA and analyzed by LC-MS/MS in an Easy-nLC II system coupled to LTQ-Orbitrap-Velos-Pro mass spectrometer (Thermo Scientific). The peptides were concentrated by reverse phase chromatography using a 0.1 mm × 20 mm C18 RP precolumn (Proxeon, Odense, Denmark), and then separated using a 0.075 mm x 100 mm C18 RP column (Proxeon) operating at 0.3 μL/min. Peptides were eluted using a 90-min gradient from 5 to 40% solvent B (Solvent A: 0.1% FA in water, solvent B: 0.1% FA and 80% ACN in water). ESI ionization was performed using a Nano-bore emitters Stainless Steel ID 30 μm (Proxeon) interface. The Orbitrap resolution was set at 30.000. Peptides were detected in survey scans from 400 to 1600 amu (1 ìscan), followed by ten data dependent MS/MS scans (Top 10), using an isolation width of 2 m/z units (in mass-to-charge ratio units), normalized collision energy of 35%, and dynamic exclusion applied during 30 s periods.

##### Design and Development of the Multiple Reaction Monitoring (MRM) Method

The MRM method was developed for research use, which aligns this work with a Tier 2 Targeted Measurement as described by Carr *et al.* (19). The target peptides were chosen based on three criteria: (1) peptides with the highest Xscore (>3) using the Proteome Discoverer 1.3 software, (2) peptides present in at least 4 of the 6 secretomes analyzed in the discovery phase and (3) peptides belonging to cartilage ECM proteins. Fifty-four peptide precursors and fragment ion masses were selected on this basis and assayed for MRM analysis. The five most intense transitions for each suitable precursor were selected based on data deposited in the MS/MS library using the Skyline software (20). Endogenous and SIS peptides were analyzed by LC-MS/MS using a nanoLC system (TEMPO, Eksigent) coupled to a 5500-QTRAP instrument (Sciex). After desalting with a C18 precolumn (5 μm, 300A, 100 μm*2 cm, Acclaim PepMap, Thermo Scientific) and a flow of 3 μl/min during 10 min, peptides were separated on C18 nanocolumns (75 μm id, 15 cm, 3 μm, Acclaim PepMap 100, Thermo Scientific) at a flow rate of 300 nl/min. The total 70 min gradient for the MRM method starts with 5% buffer B (0.1% FA in 95% ACN) for 3 min, 35% B from 3 until 45 min, 95% B for 1 min, hold for 10 min, and finally, equilibration of the column with 5% B during 15 min. The mass spectrometer was interfaced with nanospray sources equipped with uncoated fused silica emitter tips (20 μm inner diameter, 10 μm tip, NewObjective, Woburn, MA) and was operated in the positive ion mode. Skyline was used to predict and optimize collision energies (CE) and declustering potential (DP) for each peptide (20). Q1 and Q3 were set to unit/unit resolution (0.7 Da) and the pause between mass ranges was set to 3 ms. MRM analysis was conducted with up to 152 transitions per run (dwell time, 15 ms; cycle time 3 s).

For the validation analyses, 23 peptides were selected and included in the final method based on the following criteria: good signal in the MRM method, co-elution of at least 3 transitions and detection using the MIDAS workflow. With this aim, the best MRM transitions for these peptides were pooled in one scheduled-MRM method with a 45-min gradient, using retention times extracted during the assay refinement. Different detection windows were used and the signal was compared with the MRM-IDA acquisition methods. The detection window of 300 gave the best sensitivity with a time window of ±2.5 min because of the possible small differences in RT between different days. The signal was defined as the detection of all the transitions from the endogenous peptide exactly co-eluting with all the transitions from the stable isotope-labeled peptide. Table II shows the final list of peptides quantified in this work, whereas supplemental Table S1 enumerates all transitions that were monitored per peptide and the settings for their analysis.

##### Data Analysis

Peptide identification from raw data from the LTQ-Orbitrap was carried out using the SEQUEST algorithm (Proteome Discoverer 1.3, Thermo Scientific). Each MS/MS spectrum was searched in the Uniprot/Swissprot database (UniProt 2015_05 release version containing 547,599 sequences and 195,014,757 residues, with taxonomy restriction_*Homo sapiens*). The following constraints were used for the searches: no enzyme, no fixed and variable modifications and tolerances of 10 ppm for precursor ions and 0.8 Da for MS/MS fragment ions. Search against decoy database (integrated decoy approach) using false discovery rate (FDR) < 0.01. Data from the 5500 QTRAP were analyzed with ProteinPilot 4.0 (Sciex), using the Paragon algorithm as default search program using no enzyme and modifications criteria. Raw files were imported to Skyline and integration was manually inspected to ensure correct peak detection and accurate integration. After the unambiguous detection of selected peptides in the secretome samples, synthetic standard peptides were used for confirmatory analyses and quantitation, using one-point calibration to determine fold changes between the different OA samples (WZ and UZ) compared with healthy donors. The Protease Specificity Prediction Server (PROSPER) tool (21) was employed to search enzymes putatively involved in the cleavage of the endogenous peptides that had been identified in this work.

##### Statistical Analysis

A *p* < 0.05 was considered statistically significant and all statistical tests were two-sided. GraphPad Prism 5.0 (Graphpad, San Diego, CA, USA) was used to compare medians among the three different conditions of patients and controls (WZ-UZ-Control), and a Kruskal-Wallis test's multiple comparison was used. Mann-Whitney U tests were performed to evaluate the significance of discrimination between the disease classes and the control cohort. Receiver operator characteristic (ROC) analysis was performed to quantify the overall ability of a peptide to classify the tissue as OA or healthy. The ROC curves were smoothed, compared and threshold computed using the R package pROC 2018 (22).

## RESULTS

### 

#### 

##### Isolation and Identification of Endogenous Peptides Released from Articular Cartilage

**Fig. 1.**
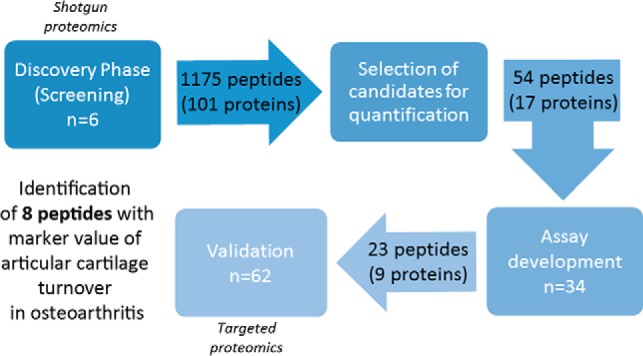
**Schematic workflow of the study.**

The experimental workflow followed for the peptidomic profiling of articular cartilage degradation and the identification of pathogenic markers in OA is summarized in Fig. 1. The studies were performed on conditioned media from human articular cartilage explants, whose characteristics were assessed by Mankin scoring (Table I). In the OA tissue, explants were obtained both from the macroscopically normal zone (unwounded zone, or UZ, with an average Mankin score of 3.52 ± 0.92) and the lesion (wounded zone, or WZ, Mankin score of 8.38 ± 1.47), to evaluate possible differences. Finally, the healthy cartilages analyzed in this work had a Mankin score of 1.76 ± 0.48.

**Table I:** Characteristics of the articular cartilage explants employed in this work. Two different explants were obtained per OA tissue (one from the UZ and another from the WZ). Thus, the number of samples analyzed is duplicated for OA cartilage

	Dx	n	% Female	Age (mean ± S.D.)	Mankin (mean)
*Screening*					
	N	2	33.3	77.33 ± 4.16	1.5
	OA	2	0	66 ± 11.31	2.5 (UZ) 7.6 (WZ)
*Total number of samples*	*6*				
*MRM Development*					
Hip	N	6	33.3	77.67 ± 8.16	1.5
	OA	5	100	82.2 ± 6.02	3.6 (UZ) 6.2 (WZ)
Knee	N	2	0	56 ± 2.83	1.5
	OA	8	62.5	82.5 ± 9.26	3.2 (UZ) 9 (WZ)
*Total number of samples*	*34*				
*Validation*					
Hip	N	13	38.46	76.38 ± 12.24	1.7
	OA	10	70	77.8 ± 9.02	3.3 (UZ) 9.3 (WZ)
Knee	N	5	40	70.6 ± 13.6	2.6
	OA	12	41.67	73.93 ± 6.97	5 (UZ) 9.8 (WZ)
*Total number of samples*	*62*				

N, healthy cartilage; UZ, Unwounded zone of OA cartilage; WZ, Wounded zone of OA cartilage.

To isolate the endogenous peptides present in the conditioned media, we explored different combinations of ultrafiltration and solid phase extraction (SPE), which led to the final protocol described in the Experimental Procedures section. Days 2 and 5 of culture were selected as the best points for the peptidomic analysis, showing the highest number of unique peptides and the lowest serum contamination in the conditioned media. The screening step led to the identification of 1175 different peptides corresponding to 101 unique proteins that were released from hip or knee articular cartilage to the conditioned media. The complete list of neopeptides that were identified, and their correspondent parent proteins, is shown in Supplemental Table S2. A higher number of peptides in OA compared with normal tissue was found, although the result was not statistically significant (*p* = 0.17). The parent proteins identified with the highest score and highest number of peptides were ECM structural constituents, such as COMP, PRELP or Fibronectin (FINC). Several of them were specifically characteristic of the articular cartilage ECM, such as COMP, Cartilage Intermediate Layer Protein 1 (CILP1) or Proteoglycan 4 (PRG4).

##### Development of Targeted Methods for the Quantitative Analysis of Endogenous Peptides Released from Articular Cartilage

The peptides that show the highest identification score (>3) in the screening phase, where identified in the majority of samples and belong to proteins expressed in articular cartilage were selected to develop a targeted analysis method based on MRM-mass spectrometry. The criteria for the selection of peptides in this phase is fully described in the methods section. Fifty-four endogenous peptides (belonging to 17 proteins) were explored for the development of the method, which was carried out using secretome samples from eleven hip and 10 knee cartilages (Table I). Then, the final MRM method was designed with the aid of SIS peptides for the detection and quantification of the 23 endogenous peptides showing the best performance (see criteria under Experimental Procedures), whose 9 parent proteins are expressed in human articular cartilage. These proteins are Matrix Gla Protein (MGP), COMP, CILP1, PRELP, Dermcidin (DCD), FINC, clusterin (CLUS), Glia Derived Nexin (GDN) and Collagen Alpha-1 (II) Chain (CO2A1). The list of endogenous peptides included in this targeted analysis is shown in Table II, and the specific transitions and MRM settings are detailed in supplemental Table S1.

**Table II:** Endogenous peptides quantified by LC-MRM in articular cartilage secretomes. Bold letters indicate the stable isotope-labeled amino acid in each peptide

Sequence	Protein Name	UNIPROT Acc No.
NANTFISPQQ**R**	Matrix Gla protein	sp P08493 MGP
NTFISPQQ**R**		
AEPGIQ**L**KAV	Cartilage oligomeric matrix protein	sp P49747 COMP
AVAEPGIQ**L**K		
V**L**NQGREIVQT		
DEGDTFPL**R**	Cartilage intermediate layer protein 1	sp O75339 CILP1
NLEPRTGF**L**SN		
STATAAQTD**L**NFIN		
DSNKIETI**P**N	Prolargin	sp P51888 PRELP
SDGVFK**P**DT		
SSD**L**ENVPH		
DLENVPHL**R**		
SSGSGPFTDVRA**A**	Fibronectin	sp P02751 FINC
TSSGSGPFTDVRA**A**		
DAVEDLESVG**K**	Dermcidin	sp P81605 DCD
ENAGEDPGLA**R**		
ASHTSDSDVPSGVTE**V**	Clusterin	sp P10909 CLUS
ASHTSDSDVPSGVTEV**V**		
GEDQYYLRVTT**V**		
SEDGTKASAATTAI**L**	Glia-derived nexin	sp P07093 GDN
AVAQTDLKEP**L**KV		
AGPPGPVG**P**AGGP	Collagen alpha-1(II) chain	sp P02458 CO2A1
AGPSGPRGPPG**P**VGP		

The area under the curve for the endogenous peptides was plotted for each peptide in samples from the UZ and WZ of OA and healthy donors. Certain peptides belonging to CILP1 (DEGDTFPLR) and PRELP (DSNKIETIPN, DLENVPHLR) were found to be mostly increased in the WZ of OA cartilages when compared with UZ and healthy donors. A representative example of the chromatograms obtained with this analysis can be seen for the peptide DSNKIETIPN in supplemental Fig. S1. To confirm these results and normalize the data, we developed a scheduled MRM method and incorporated peptides labeled with heavy stable isotopes as internal standards for the quantification.

##### Quantification of Endogenous Peptides in Cartilage Secretomes

**Fig. 2.**
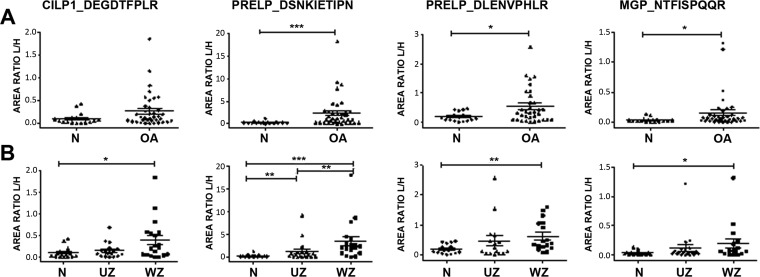
**Differential endogenous peptides released from osteoarthritic articular cartilage.** Scattering plots representing the different abundance of each peptide in the cartilage secretomes. *A*, Comparison between OA (*n* = 44) and normal tissue (*n* = 18). *B*, OA samples were classified into those from the unwounded zone of the tissue (UZ, *n* = 22) and from the wounded (WZ, *n* = 22). The results are expressed as area ratios (light/heavy, L/H). Data were analyzed using Mann-Whitney test and plotted as means ± standard error of the mean (SEM) for each condition. *, *p* < 0.05; **, *p* < 0.005; ***, *p* < 0.0005.

The validation study was carried out using the scheduled MRM method and stable isotope labeled peptide standards on 62 secretome samples obtained from hip (*n* = 33) and knee (*n* = 29) cartilage. All the quantification data (expressed as peak area ratios of light/heavy peptides) from the peptides in the secretome of different zones of OA cartilage (UZ and WZ) and healthy donors in the different joints are showed in Supplemental Table S3. After statistical analysis of the results, four endogenous peptides were found to be differentially released from OA cartilage compared with healthy tissue with a significant *p* value. Among these, two peptides from PRELP (DSNKIETIPN and DLENVPHLR) and one from MGP (NTFISPQQR) were differentially released independently of the OA cartilage zones (Fig. 2*A*). Furthermore, the same tendency was found in the OA WZ compared with control donors for these peptides and the peptide DEGDTFPLR from CILP1. All of them were found increased in the OA WZ *versus* healthy cartilage (Fig. 2*B*). Finally, the peptide DSNKIETIPN (PRELP) was differentially released in the UZ compared with normal cartilage, and between the two OA cartilage zones.

##### Differential Release of Endogenous Peptides from Knee and Hip Articular Cartilages

The targeted peptide quantification evidenced a differential release of certain neopeptides depending on the joint that was studied (*p* < 0.05), which are shown in supplemental Table S4 and supplemental Fig. S2. In all cases, the release was higher from the knee tissue. Although no precision measurements could be performed because of the limiting amount of peptides that could be obtained in the samples, which restricted the analysis to one injection per sample, we did observe very reproducible retention times (%CV<10%) for all the neopeptides quantified in the targeted analysis (supplemental Table S5).

Comparison of the conditioned media from all knee (*n* = 29) and hip (*n* = 33) cartilage samples demonstrated the increased release from knee of endogenous peptides corresponding to the MGP (NANTFISPQQR and NTFISPQQR), COMP (AEPGIQLKAV) and PRELP (DSNKIETIPN), with fold changes ranging from 2.29 to 5.11 (supplemental Fig. S2*A*). In OA cartilage, the peptide AEPGIQLKAV (COMP) has a remarkable 8-fold change ratio higher in knee *versus* hip, whereas DSNKIETIPN from PRELP, GEDQYYLRVTTV and ASHTSDSDVPSGVTEV from CLUS also showed significant differences (supplemental Fig. S2*B*). Considering only the healthy tissues (knee *n* = 5 and hip *n* = 13), one peptide was increased in the knee samples (NTFISPQQR, from MGP) with a fold ratio of 3.54 (supplemental Fig. S2*C*).

**Fig. 3.**
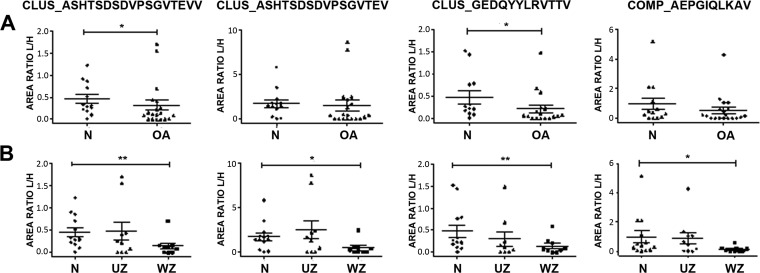
**Differential endogenous peptides released from hip articular cartilage.** Scattering plots showing the abundance of each peptide in hip cartilage secretomes. *A*, Comparison between OA (*n* = 20) and normal tissue (*n* = 13). *B*, OA samples were classified into those from unwounded zones (UZ, *n* = 10) or wounded zones (WZ, *n* = 10). The results are expressed as area ratios (light/heavy, L/H). Data were analyzed using Mann-Whitney test and plotted as means ± SEM for each condition. *, *p* < 0.05; **, *p* < 0.005.

Given these joint-characteristic profiles, the differences in the release of peptides were examined independently in hip and knee samples. In hip, two peptides from CLUS were increased in the conditioned media of healthy cartilage compared with OA tissue: ASHTSDSDVPSGVTEVV and GEDQYYLRVTTV (Fig. 3*A*). When the different zones in the diseased cartilage were taken together (Fig. 3*B*), these two peptides showed a significant lower release from the wounded zone of the tissue (WZ). The same happens with another peptide from CLUS, ASHTSDSDVPSGVTEV, and the peptide AEPGIQLKAV from COMP.

**Fig. 4.**
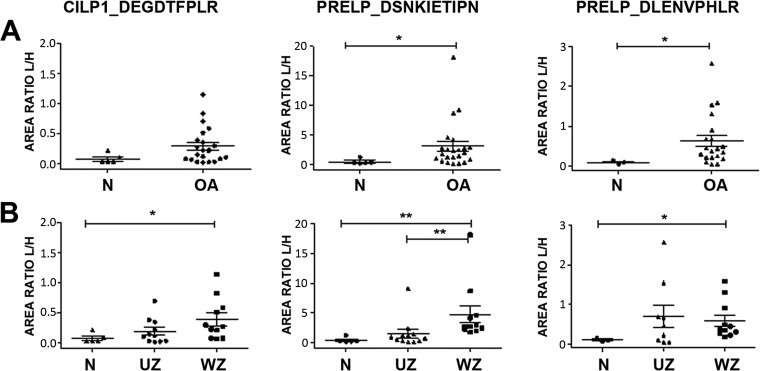
**Differential endogenous peptides released from knee articular cartilage.** Scattering plots showing the abundance of each peptide in knee cartilage secretomes. *A*, Comparison between OA (*n* = 24) and normal tissue (*n* = 5). *B*, OA samples were classified into those from unwounded zones (UZ, *n* = 12) or wounded zones (WZ, *n* = 12). The results are expressed as area ratios (light/heavy, L/H). Data were analyzed using Mann-Whitney test and plotted as means ± SEM for each condition. *, *p* < 0.05; **, *p* < 0.005.

In knee samples, two endogenous peptides from PRELP were significantly increased in the conditioned media of OA tissue: DSNKIETIPN and DLENVPHLR (Fig. 4*A*). Considering the two zones of OA tissue separately, these two peptides showed an enhanced release specifically from the WZ (Fig. 4*B*). Interestingly, the peptide DSNKIETIPN exhibited the most significant differences, which were also detectable in samples from the UZ of OA tissue. The peptide DEGDTFPLR from CILP1 displayed a similar tendency.

##### Value of the Identified Peptides as Biomarkers of Articular Cartilage Degradation

**Fig. 5.**
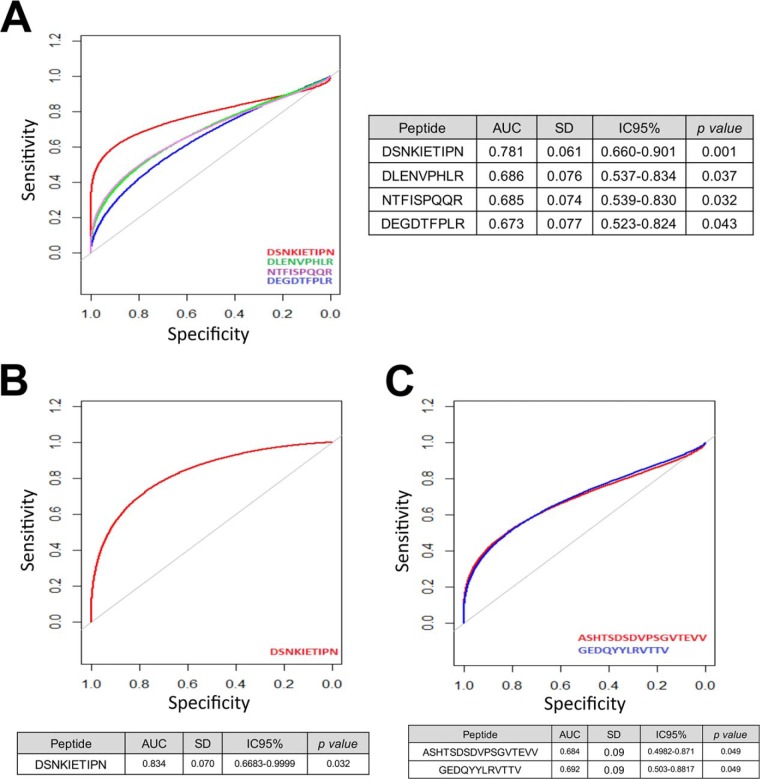
**Receiver operator characteristic (ROC) curves of the biomarker peptides identified in this work.** *A*, The release of four peptides discriminates OA *versus* healthy articular cartilage with significant *p* value (*p* < 0.05), *B*, The peptide DSNKIETIPN from prolargin differentiates knee OA from healthy tissue, and *C*, Two peptides from clusterin discriminate hip OA from healthy tissue.

To evaluate the putative biomarker value of the endogenous peptides that have been identified, an analysis by receiver operator characteristic (ROC) curves was performed. As illustrated in Fig. 5*A*, the peptide DSNKIETIPN showed an area under the curve (AUC) of 0.781 [IC 95%: (0.660–0.901), *p* = 0.001], being the best candidate to discriminate healthy *versus* OA tissue independently of the target joint. Considering only the knee, the AUC of this peptide increased up to 0.834 (Fig. 5*B*). On the other hand, two peptides from CLUS (ASHTSDSDVPSGVTEVV and GEDQYYLRVTTV) displayed significant AUCs when analyzing the hip tissue exclusively (Fig. 5*C*).

Finally, we also performed this analysis by splitting the OA tissue in zones (supplemental Fig. S3). In this case, again the best results were obtained for the peptide DSNKIETIPN in knee, showing a good biomarker value (AUC = 0.783) in OA but macroscopically normal cartilage. Comparing healthy knee tissue with the damaged zones of knee OA, this AUC increased up to 0.891. In hip, the performance of GEDQYYLRVTTV was worse, but still significant (AUC normal *versus* WZOA = 0.761).

## DISCUSSION

Peptides are constantly generated *in vivo* either by active synthesis and proteolytic processing of larger precursor proteins, often yielding protein fragments that mediate a variety of physiological or pathological functions. Given that abnormal proteolysis is a hallmark of various diseases, many studies turned to focus on the peptidome (23) as a source of biomarkers. The investigation of peptides in a system-wide manner could facilitate the identification of potential biomarkers, the identification of protease-substrate relationships and the profiling of pathological degradation processes.

Analyses have been performed under different conditions in order to elucidate the regulatory biological phenomenon of neopeptides in OA. On the one hand, some studies were developed to identify and characterize individual enzyme-generated cleavage products *in vitro*, leading for instance to the identification of specific aggrecanase neoepitopes such as ARGSV and NITEGE (24). On the other hand, some works were particularly focused on the identification of neopitopes in this case from specific cartilage ECM-related proteins, such as FINC (25) or COMP (26). In contrast to these, the present study provides a hypothesis-free, shotgun analysis to search for novel neopeptides that could be differentially released from OA cartilage because of the disease process.

Our two-step peptidomic analysis started with a first discovery phase identifying 1175 different peptides corresponding to 101 unique proteins. This is, to our knowledge, the deepest characterization of cartilage neopeptides. Interestingly, in general we detected more peptides and with higher signals in secretomes from knee samples than from hip (supplemental Figs. S1 and S2), which depicts the differences between these two joints and also indicates a higher turnover in the knee that could not been revealed in previous proteomic analyses performed directly on the tissue (3, 27). Data mining showed that most of the identified proteins were cartilage ECM proteins or proteins with well-established matrix functions, such as collagens and proteoglycans. Although some of the parental proteins of many of these neopeptides have been reported for the first time in cartilage-derived samples (such as salivary acidic proline-rich phosphoprotein 1/2) many of them had been previously associated with OA: type II collagen, proteoglycan 4, FINC or COMP. Notably, our list of neopeptides includes the detection of previously known OA biomarkers, such as CTXII (peptides GPDPLQYMRA, DPLQYMRA and SAFAGLGPRE, from the C-telopeptide fragment of type II collagen). Altogether, this further evidences the usefulness of secretome analysis as a source of cartilage-characteristic biomarkers (15, 18, 28–29).

Next, in a second validation step, we selected a panel of these endogenous peptides and developed a targeted MRM-based method for their quantification in secretomes. This method was then applied for an exhaustive analysis on 62 secretomes from articular cartilage, which allowed to obtain statistically significant results of the differences. Eight endogenous peptides were found to be differentially released from OA compared with healthy tissue. The metrics obtained in this study are summarized in Table III.

**Table III:** Endogenous peptides identified as putative OA biomarkers in human articular cartilage. Numbers show the p value calculated in each case

Peptide	Protein	N vs OA	N vs UZ	N vs WZ	UZ vs WZ
*Peptide markers of OA*					
DEGDTFPLR	CILP1			0.0233	
DSNKIETIPN	PRELP	0.0008	0.049	0.0001	0.0094
DLENVPHLR	PRELP	0.0376		0.0047	
NTFISPQQR	MGP	0.0327		0.0202	
*Peptide markers of Knee OA*					
DEGDTFPLR	CILP1			0.0235	
DSNKIETIPN	PRELP	0.0226		0.0022	0.0012
DLENVPHLR	PRELP	0.04		0.0127	
*Peptide markers of Hip OA*					
ASHTSDSDVPSGVTEVV	CLUS	0.0383		0.0076	
ASHTSDSDVPSGVTEV	CLUS			0.0162	
GEDQYYLRVTTV	CLUS	0.0237		0.0277	
AEPGIQLKAV	COMP			0.0194	

N, healthy tissue; UZ, unwounded zone of OA cartilage; WZ, wounded zone of OA cartilage.

Because neopeptides are the result of protease performance on the tissue, their concentrations in the medium may be altered by the activity of proteases, digesting proteins at specific amino acid locations. Thus, depending on the protease type and their corresponding site of digestion, specific neopeptides can be either generated (increased) or degraded (decreased) in OA or N samples. Remarkably, we found decreased amounts of three neopeptides from CLUS and one from COMP in hip OA samples (Fig. 3). This is in accordance with the disease-related significant decrease of these two proteins in articular cartilage that has been described recently (3). CLUS, also known as Apolipoprotein J, is a secreted protein that regulates apoptosis and inflammation. A few studies have observed elevated CLUS in cartilage and synovial fluid in early OA (30, 31). Furthermore, increased CLUS levels in SF and serum showed statistically significant associations with joint space narrowing after adjustment for age and sex (32). However, IL-1α-stimulated cartilage explants have shown to produce decreased levels of CLUS compared with untreated cartilage (3, 33). An analogous discrepancy happens with COMP: although this protein is decreased in knee and hip OA articular cartilage (*p* = 0.007) (3), it is well known that its elevated levels in serum are associated with OA severity (7, 34). An explanation for this might be that these higher levels of CLUS and COMP in OA SF and plasma could represent the activation of a compensatory, but ultimately ineffective, protective pathway.

In the knee, we observed the disease-related increase of one neopeptide from CILP1 and two from PRELP. This increase was significant from the WZ zones of the tissue in all cases, but in the case of the peptide DSNKIETIPN from PRELP it was also detectable in the macroscopically normal zone. This result suggests that this peptide may have value as marker of early OA. Furthermore, the ROC analysis showed the best results for this peptide (Fig. 5), with and AUC of 0.834 for the classification of the tissue as OA or healthy, with a high specificity (0.821) for OA. Interestingly, DSNKIETIPN was identified in a previous study as the relatively most abundant peptide from an *in vitro* digestion with ADAMTS4 (20). The contribution of the aggrecanases ADAMTS4 and ADAMTS5 to cartilage destruction in OA has been widely established (35, 36), although it has not been resolved completely. PRELP is a small leucine-rich proteoglycan highly abundant in cartilage (37, 38) that binds the basement membrane heparan sulfate proteoglycan perlecan through its N-terminal region, and collagens (type I and II) through its 12 leucine-rich repeat (LRR) domains. An increase in DSNKIETIPN, localized in the 7th LRR domain of the protein, denotes PRELP breakage with a loss of half its LRR domains for collagen binding. Thus, the statistically significant increase of this neopeptide in OA cartilage that we demonstrate in the present work depicts the role of PRELP as mediator of ADAMTS4 catabolic effects in articular cartilage.

Finally, it is important to highlight that most of the neopeptides that yielded significant results could be proposed to be markers of advanced OA, because they display statistical differences only between N and WZOA samples. As mentioned before, the DSNKIETIPN neopeptide from PRELP was the only one exhibiting a potential marker value for early OA. However, these considerations might be taken with caution considering the *in vitro* model of cartilage explants that has been employed in this screening study. Given the complex nature of OA, which involves not only articular cartilage but also other joint tissues such as the synovial membrane or the subchondral bone (1), it may be risky to associate at this point the identified neopeptides with specific stages of disease severity. In order to robustly assess the correlation of these molecules with either structural features and/or clinical symptoms in OA patients, it would be necessary to analyze samples from large, well-characterized cohorts. With this aim, the first step would be to develop a more sensitive technique that enables the detection of these neopeptides in biological fluids. For instance, monitoring the detected peptides by neopeptide-targeted ELISAs (39) or other emerging techniques that can combine the sensitivity of immuno-affinity assays with the selectivity of MS analysis, such as SISCAPA-MRM (40) or immuno-MALDI techniques (41).

In summary, we have performed a peptidomic analysis for the discovery and validation of novel neopeptides associated with the degradation of human articular cartilage in osteoarthritis. This work has enabled not only to obtain an exhaustive neopeptidomic profile of this tissue, but also the identification and validation of a panel of eight differential endogenous peptides that are released in the pathogenic process. The peptide DSNKIETIPN, from Prolargin, showed the best metrics as a biomarker of OA cartilage, proving to be the most promising candidate for the development of assays aimed at its detection and quantification in biological fluids.

## Data Availability

The mass spectrometry peptidomic data have been deposited to the ProteomeXchange Consortium via the PRIDE partner repository with the data set identifier PXD011800. Data obtained in the MRM-based analysis have been uploaded to PeptideAtlas and can be accessed via http://www.peptideatlas.org/PASS/PASS01294. All chromatograms employed for quantification are available in Panorama Public, with the access URL: https://panoramaweb.org/NeopeptidesOAcartilage.url.

## Supplementary Material

Supplemental Data

Supplementary Table S1

Supplementary Table S2

Supplementary Table S3

Supplementary Table S4

Supplementary Table S5

## Footnotes

^1^ The abbreviations used are:

OA: osteoarthritis
CILP1: cartilage intermediate layer protein 1
CLUS: clusterin
CO2A1: collagen alpha-1 (II) chain
COMP: cartilage oligomeric matrix protein
ECM: extracellular matrix
FINC: fibronectin
GDN: glia-derived nexin
MGP: matrix Gla protein
PRELP: prolargin
UZ: unwounded zone (of OA tissue)
WZ: wounded zone (of OA tissue).
