# Supplementary material for: Analysis of Endogenous Peptides Released from Osteoarthritic Cartilage Unravels Novel Pathogenic Markers
Source: Mol Cell Proteomics. 2019 Jul 27;18(10):2018–28. doi: 10.1074/mcp.RA119.001554 (PMC6773562; doi:10.1074/mcp.RA119.001554)
Supplement: Supplemental Data [file 152705_1_supp_359569_p8bxk8.pdf]

Supplemental Data

Supplementary Tables

Supplementary Table S1. Targeted proteomics design. MRM mass spectrometry transitions analyzed in this work, and settings for their analysis.

Supplementary Table S2. Full results from the discovery phase. A) Endogenous peptides identified in the secretomes of human articular cartilage. **B)** Unique proteins corresponding to the endogenous peptides identified in this work.

Supplementary Table S3. Quantification data obtained for the panel of peptides analyzed by MRM mass spectrometry. Results are expressed in peak area ratios of abundance (light/heavy peptides), with a confidence level of p<0.05*.

Supplementary Table S4. Fold changes of endogenous peptides differentially released from knee and hip articular cartilage with a significant p-value (<0.05). Data were obtained using the MS stats tool from Skyline software.

Supplementary Table S5. Coefficient of variation (%CV) of the retention times (RT) obtained for each peptide by SRM analysis on human articular cartilage secretomes.

Supplementary Figures

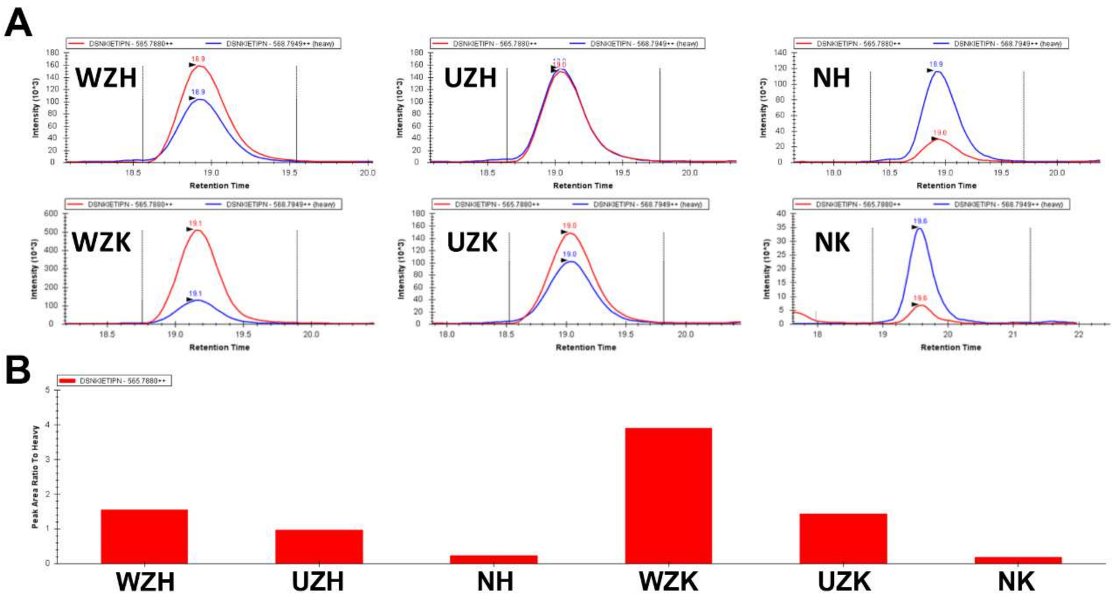

Supplementary Figure S1. Multiple Reaction Monitoring (MRM) - mass spectrometry quantification of endogenous peptides. A) Representative chromatograms of the endogenous peptide PRELP_DSNKIETIPN in a pool of secretome

samples (n=3) from hip (upper row) and knee (lower row). The endogenous peptides (light) are represented in red, whereas the heavy peptide standards (SIS) are displayed in blue. The amount of SIS spiked into each sample was kept constant. B) Chart plot representing the peak area ratio normalized to the heavy peptide standard for each type of sample. WZH, wounded zone from hip OA; UZH, unwounded zone from hip OA; NH, healthy hip; WZK, wounded zone from knee OA; UZK, unwounded zone from knee OA; NK, healthy knee.

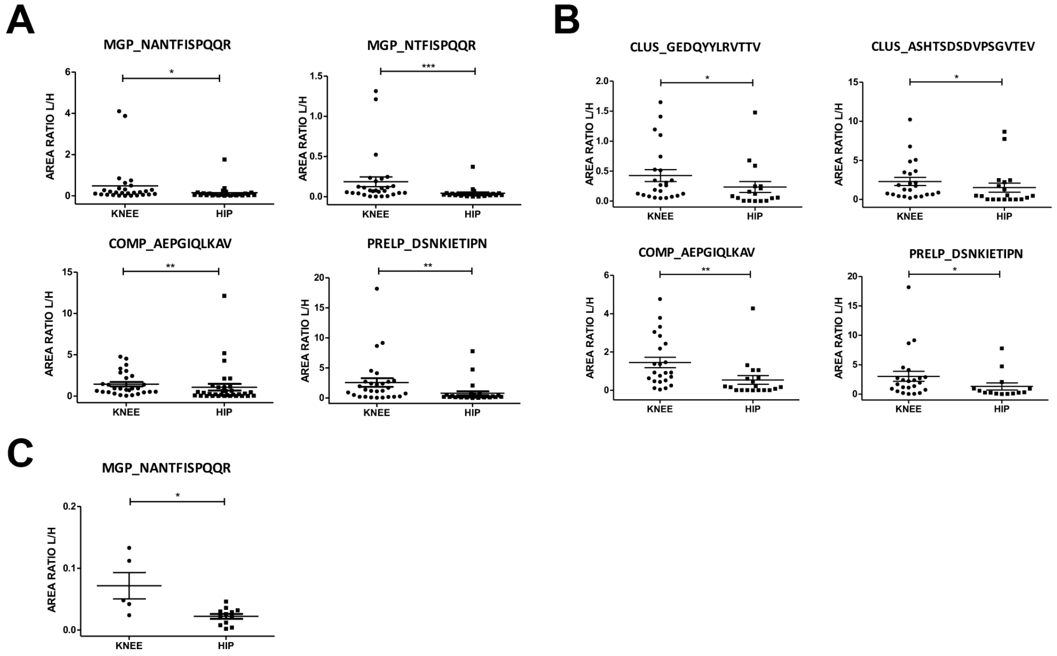

Supplementary Figure S2. Differential release of endogenous peptides from hip and knee articular cartilages. Scattering plots showing the distribution of the Area light/heavy (L/H) ratios of representative endogenous peptides. The data were analyzed using Mann-Whitney test and plotted as means ± SEM for each condition. A) Knee (n=29) vs hip (n=33), B) OA knee (n=23, 12 WZ and 11 UZ) vs OA hip (n=20, 10 WZ and 10 UZ), and C) Healthy knee (n=5) vs healthy hip (n=13). *p* < 0.05, p**<0.005 p***<0.0005.*

A

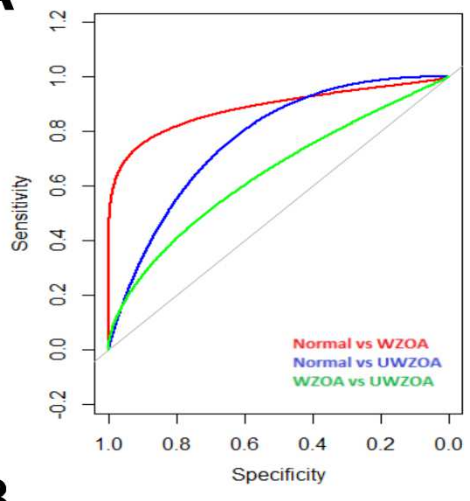

Peptide	Multi-class AUC
**DSNKIETIPN**	0.7702

Peptide	AUC	*p value*	*power*
DSNKIETIPN Normal vs WZOA	0.8909	0.044	0.812
DSNKIETIPN Normal vs UWZOA	0.7833	0.05	0.459
DSNKIETIPN WZOA vs UWZOA	0.530	0.049	0.130

B

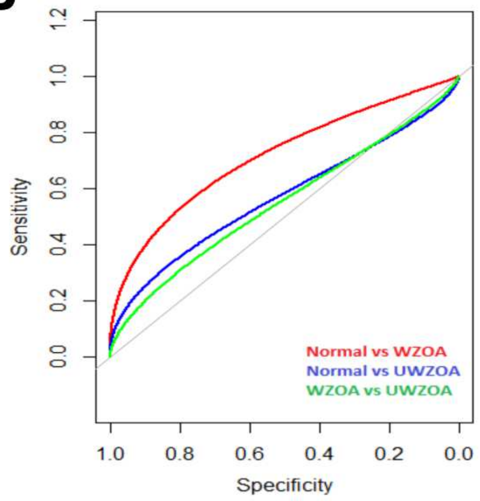

Peptide	Multi-class AUC
**GEDQYYLRVTTV**	0.6133

Peptide	AUC	*p value*	*power*
GEDQYYLRVTTV Normal vs WZOA	0.7607	0.049	0.605
GEDQYYLRVTTV Normal vs UWZOA	0.6239	0.049	0.176
GEDQYYLRVTTV WZOA vs UWZOA	0.6049	0.050	0.127

Supplementary Figure S3. Receiver operator characteristic (ROC) curves of the best biomarker peptides differentiating disease and zone in knee (A) or hip (B) articular cartilage. The inset tables show the metrics obtained for each peptide in normal (healthy), unwounded (UW) or wounded (W) zones of OA tissue from each joint.
